# Patent foramen ovale: life-saving, but breath-taking in fulminant pulmonary embolism

**DOI:** 10.1093/ehjcr/ytaf157

**Published:** 2025-04-02

**Authors:** Kathrin Reuthner, Thomas Müller, Alexander Dietl

**Affiliations:** Department of Internal Medicine III, University Hospital Regensburg, Franz-Josef-Strauß-Allee 11, Regensburg D-93053, Germany; Department of Internal Medicine II, University Hospital Regensburg, Franz-Josef-Strauß-Allee 11, Regensburg D-93053, Germany; Department of Internal Medicine II, University Hospital Regensburg, Franz-Josef-Strauß-Allee 11, Regensburg D-93053, Germany

**Figure:**
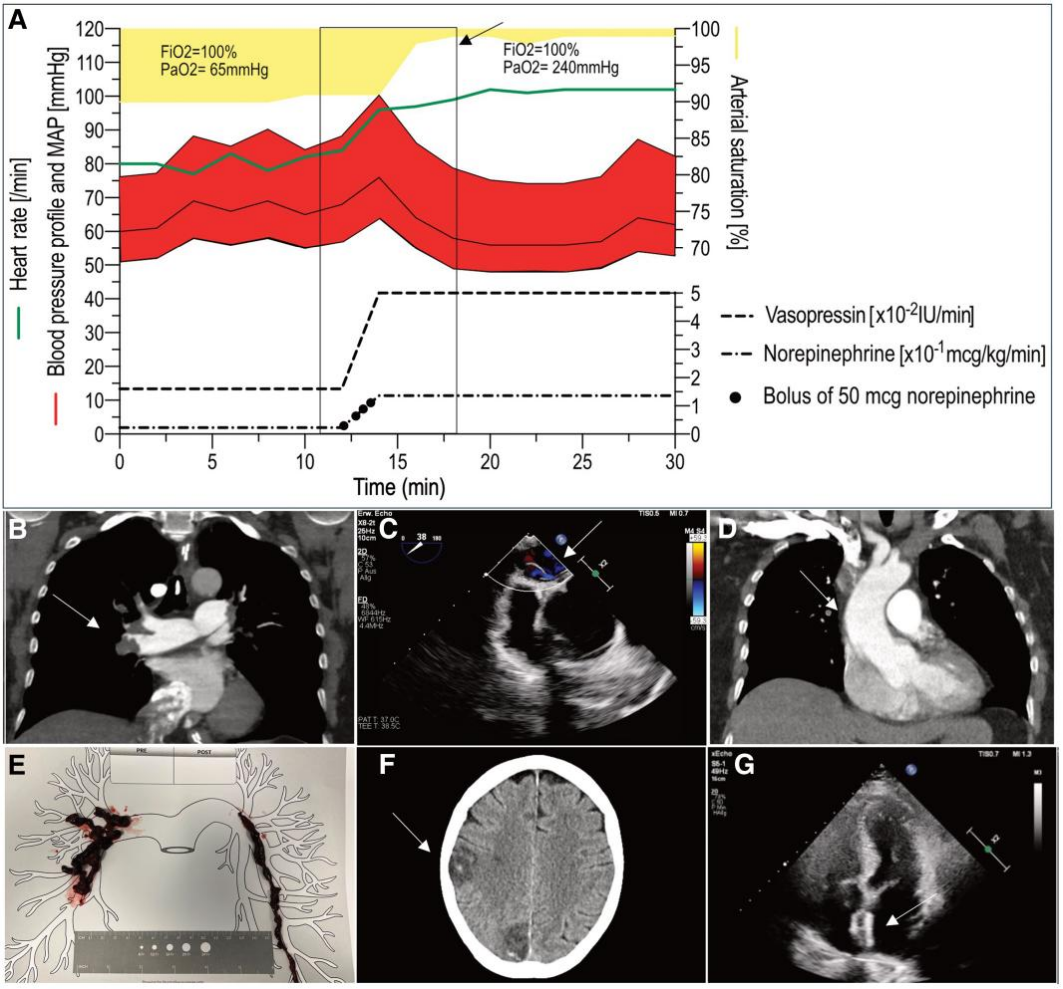


Two days after knee surgery, a 61-year-old female patient developed acute dyspnoea followed by hypoxaemic respiratory failure. The patient had no medical history. A week before, she had been able to cycle to work. A CT scan revealed bilateral central pulmonary artery emboli despite the administration of thromboprophylaxis (*Panel B*). In critical hypoxaemic failure despite non-invasive ventilation (NIV), bronchoscopic intubation was performed in upright position through the oronasal NIV mask. After the upper body was lowered, there was a sudden haemodynamic collapse accompanied by a significant improvement in oxygen saturation (*Panel A*). With persistent hypoxaemia, a right-to-left atrial shunt was suspected. In line, transthoracic echo revealed a jet on colour Doppler. Transoesophageal echocardiography provided further information on the morphology, showing a patent foramen ovale (PFO) (*Panel C*). The thin, rather mobile atrial septum was displaced to the left atrium resulting from high right atrial pressure. When pulmonary embolism initially increased pressures in pulmonary circulation, the right-to-left shunt most probably had prevented the right heart from acute failure. Lowering the upper body in the presence of a PFO as well as aortic elongation (*Panel D*) reduces the right-to-left shunt volume in orthodeoxia syndrome. In our case, reduced right-to-left shunt volume improved oxygenation but unmasked the fulminant obstructive shock. As progressive shock could not be stabilized by conventional therapy, veno-arterial extracorporeal membrane oxygenation (VA-ECMO) was established. Ischaemic stroke within previous six months was a contraindication for systemic fibrinolysis. Due to acute haemodynamic instability, interventional aspiration thrombectomy was performed and removed a large amount of thrombus material (*Panel E*). After further clinical stabilization, the ECMO could be removed after 5 days. On Day 8, the patient was extubated. Patent foramen ovale was most likely causal for multi-temporal, subacute, and older cerebral ischaemic insults, which were detected by CT (*Panel F*). After interdisciplinary assessment and shared decision-making with patients’ relatives, early transcatheter closure was performed (*Panel G*). The coagulation disorder assessment did not reveal thrombophilia. After neurological rehabilitation, the patient was discharged with oral anticoagulation and is now an active part in her family’s daily life again.

## Data Availability

The data underlying this article will be shared on reasonable request to the corresponding author.

